# Assessment of Controlled Substance Distribution to U.S. Veterinary Teaching Institutions From 2006 to 2019

**DOI:** 10.3389/fvets.2020.615646

**Published:** 2020-12-18

**Authors:** Brian J. Piper, Kenneth L. McCall, Lori R. Kogan, Peter Hellyer

**Affiliations:** ^1^Department of Medical Education, Geisinger Commonwealth School of Medicine, Scranton, PA, United States; ^2^Center for Pharmacy Innovation and Outcomes, Geisinger Precision Health Center, Forty Fort, PA, United States; ^3^Department of Pharmacy Practice, University of New England, Portland, ME, United States; ^4^Department of Clinical Sciences, College of Veterinary Medicine and Biomedical Sciences, Colorado State University, Fort Collins, CO, United States

**Keywords:** amphetamine, fentanyl, codeine, methadone, pentobarbital, veterinarian, barbiturates, opioids

## Abstract

**Objective:** To evaluate the changing pattern of distribution of Schedule II and III opioids, barbiturates, and stimulants to veterinary educational institutions in the United States.

**Design:** Longitudinal study.

**Sample:** Veterinary teaching institutions that use Schedule II and III drugs.

**Procedures:** Distribution of controlled substances to veterinary teaching institutions was obtained from the Drug Enforcement Administration's Automated Reports and Consolidated Orders System (ARCOS) for opioids (e.g., methadone, fentanyl, codeine), barbiturates (pentobarbital, butalbital), and stimulants (amphetamine, methylphenidate, lisdexamfetamine) from 2006–2019. Opioids were converted to their morphine milligram equivalents (MME) for evaluation over time.

**Results:** Controlled substance distribution to veterinary schools exhibited dynamic, and agent specific, changes. The total MME for 11 opioids peaked in 2013 and decreased by 17.3% in 2019. Methadone accounted for two-fifths (42.3%) and fentanyl over one-third (35.4%) of the total MME in 2019. Pentobarbital distribution was greatest by weight of all substances studied and peaked in 2011 at 69.4 kg. Stimulants underwent a pronounced decline and were very modest by 2014.

**Conclusions and Clinical Relevance:** Opioids by total MME in veterinary teaching practice have undergone more modest changes than opioids used with humans. Hydrocodone, codeine and recently fentanyl use have declined while methadone increased. Stimulant distribution decreased to become negligible. Together, this pattern of findings warrant continued monitoring.

## Introduction

The United States continues to be adversely impacted by an iatrogenic opioid epidemic with overdoses continuing to increase (1–3). This has prompted heightened vigilance of Schedule II prescription patterns as these agents have legitimate medical uses (4) but also misuse potential (5, 6). Analysis of records submitted to the Drug Enforcement Administration (DEA) reveal that prescription opioids peaked in 2011 (7). The use of opioids for pain subsequently declined (8) while substances used for opioid use disorder like buprenorphine increased (9). The DEA production quotas from 2010 to 2020 decreased by 22.4% for codeine, 35.9% for oxycodone, 36.7% for hydrocodone, and 43.1% for fentanyl relative to only 7.7% for pentobarbital (10, 11). At the same time, there has been an increased attention to the development of more cautious prescribing practices by general practitioners (12), surgeons (13), emergency medicine specialists (14), and dentists (15). Similar efforts for veterinarians, however, have been lacking (16, 17).

Animal pain, however, is a major consideration for many of the one-hundred and thirteen thousand veterinarians practicing in the US (18). Although many authors were calling for pain to be considered the 4th vital sign prior to 2007, pain was first categorized as such by the American Animal Hospital Association in 2007 (19). Yet, four-fifths (83.3%) of Veterinary Information Network (VIN) members report difficulties obtaining opioids in the past 6-months for use in their clinics (17). In addition, there have been increasing concerns about the practice of “vet shopping” or diverting opioids from pets to their owners (17, 20–23). Attempts to control or monitor this behavior is challenged by a lack of homogenous state laws. For example, one-third of US states do not permit veterinarians to conduct Prescription Drug Monitoring Programs queries (24).

Veterinary educators have an opportunity to contribute to curbing this public health crisis. Despite the importance of this topic, most veterinarians report feeling they lack the knowledge and education needed to understand what role they can play in helping control this issue. Veterinary curricula do not typically cover this topic, evidenced by the fact that 73% of surveyed veterinarians rate their veterinary school training on opioid abuse/misuse as only fair, poor or absent (22).

The primary objective of this report was to characterize changes in opioid distribution over the last 14-years within teaching institutions. A secondary goal was to examine other controlled substance classes including barbiturates and stimulants.

## Materials and Methods

### Procedures

Manufacturers and distributors report controlled substances transactions to the DEA's Diversion Control Division and this information is publicly available in annual reports (25). Data was extracted from report seven of the DEA's Automated Reports and Consolidated Ordering System (ARCOS). ARCOS distribution data was previously validated by comparing oxycodone results to that of a state Prescription Drug Monitoring Program which showed a satisfactory (*r* = +0.985) correlation (7). Similarly, a high agreement with California's Prescription Drug Monitoring Program was found when use was classified into high vs. low by 3-digit zip code for stimulants (26). The “teaching institutions” business activity provides information on Schedule II and III substances that are dispensed to non-humans to ~200 registrants nationally. Teaching institution was defined as “A physical location where inpatient, outpatient, or emergency medical services are not provided to human patients, but where medicine is taught under the authority of a state accredited college or university”. This definition does not include individual practitioners licensed to practice medicine in a state (27).

ARCOS has been employed in many state (16), territory (28), and national (7–9) investigations and reports nationally and statewide on agents by weight (>0.1 g). ARCOS reporting was uniform from 2006 to 2017 but beginning in 2018, barbiturates and infrequently distributed stimulants (e.g., cocaine) as well as opioids (e.g., fentanyl analogs) were no longer reported. Procedures were deemed exempt by the IRB of the University of New England.

### Data Analysis

The year with the largest weight for each drug was determined. Opioids were converted to Morphine Mg Equivalents (MME) using the following multipliers: buprenorphine 10, codeine 0.15, fentanyl 75, hydrocodone 1, hydromorphone 4, meperidine 0.1, methadone 8, morphine 1, oxycodone 1.5, oxymorphone 3, and tapentadol 0.4 based on the US Centers for Medicare and Medicaid Services (29) and published research (7). For example, the weight (g) of the very potent fentanyl distributed in 2019 (14.01) was multiplied by 75 (1,050.75 MME). Figures were prepared with Graph Pad Prism, version 6.

## Results

**Figure 1:**
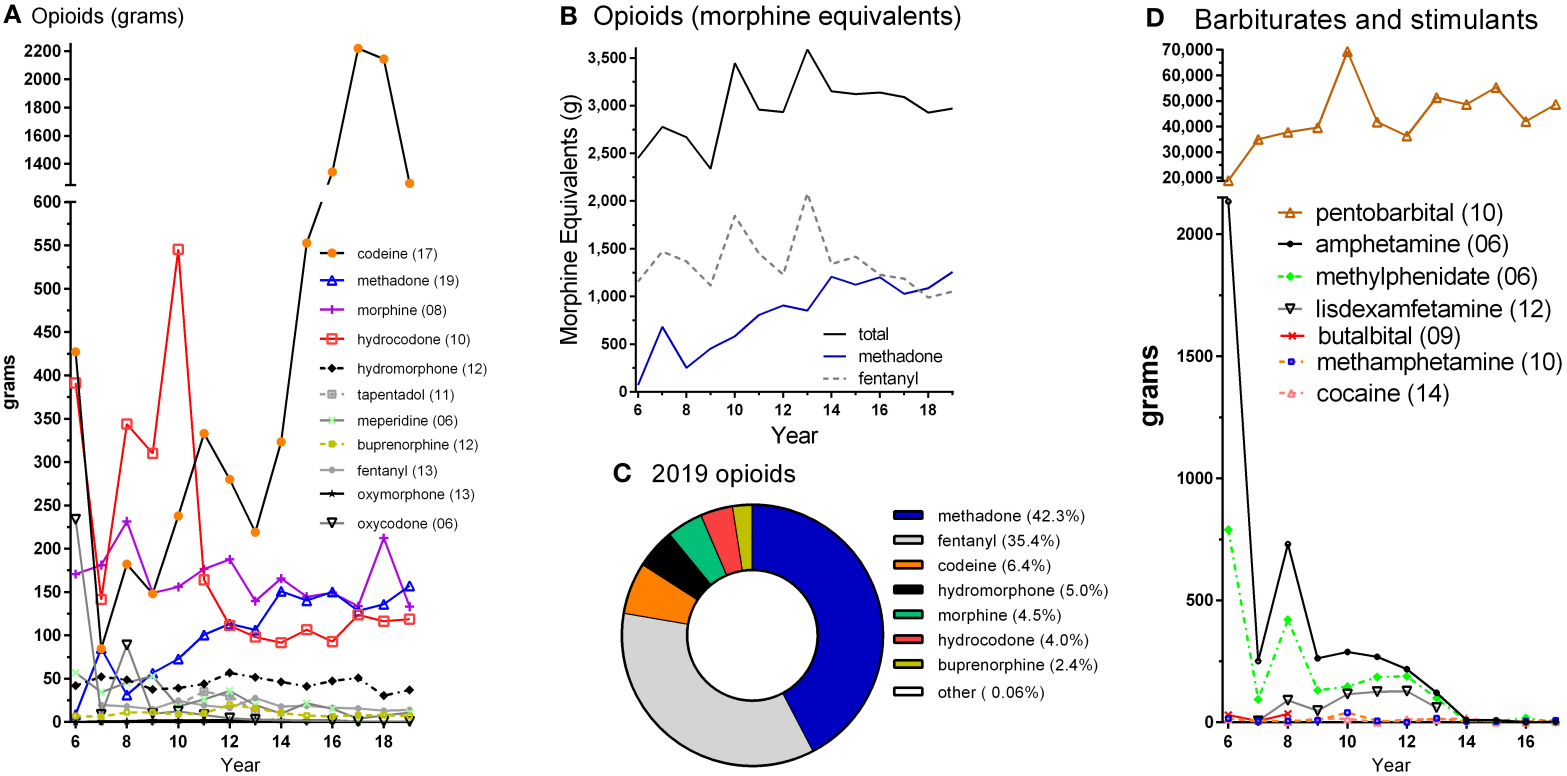
Controlled substances distribution by weight **(A,D)** and morphine equivalents **(B,C)**, by year in US veterinary teaching institutions as reported to the Drug Enforcement Administration's Automation of Reports and Consolidated Ordering System. The peak year is shown in parentheses in A and D. “Other” includes meperidine and oxymorphone in C.

Figure 1A shows opioid distribution to veterinary teaching institutions from 2006 to 2019. Codeine was the predominant Schedule II/III opioid by weight. Codeine peaked in 2017 but decreased by 43.1% in 2019. Hydrocodone reached a maximum in 2010 and then underwent a pronounced decline in 2011. Oxycodone went from 233.1 g in 2006 to <5 g beginning in 2012. Meperidine in 2019 was less than one-fifth (19.1%) of its 2006 distribution. The distribution of morphine, hydromorphone, and oxymorphone were comparatively stable during this period. The fentanyl analog remifentanil was highest at 380 mg in 2008, sufentanil at 50 mg in 2009, and alfentanil at 40 mg in 2013 (not shown).

Figure 1B depicts the total MME for 11 opioids over this 14-year interval. The top two opioids by MME are also shown. Methadone increased 17.8-fold while fentanyl was relatively consistent (−9.2%) from 2006 to 2019. Figure 1C illustrates the percent of the total MME for each opioid in 2019. Methadone and fentanyl together accounted for over three-quarters (77.7%) of the total.

Figure 1D shows the distribution of barbiturates and stimulants. Pentobarbital was at least 8-fold more common by weight than any opioid (Figure 1A) or stimulant. With the exception of 2010, the general pattern was of a gradual increase over time in pentobarbital. The high value in 2010 (69.4 kg) should be viewed with caution as New York (22.6 kg) and New Jersey (23.5 kg) were each elevated about 100-fold relative to 2009 and 2011. Amphetamine decreased 88.2% from 2006 until 2017. Methylphenidate was at 789 g in 2006 and declined to <0.5 g in 2017. Cocaine distribution never exceeded 20 g per year. Lisdexamfetamine levels were so low as to not warrant reporting beginning in 2014. Further information for stimulants and opioids for 2001 until 2005 may be found in Supplementary Figure 1.

Further regional analyses were completed on the predominant opioid by MME and the most common barbiturate. Methadone distribution in 2019 was limited to only six states. Three-states each accounted for about a quarter of the total (MI: 28.2%, OK: 25.3%, PA: 20.6%) methadone with Texas (14.5%), Illinois (8.2%), and Virginia (3.2%) responsible for the remainder. Distribution of Schedule II pentobarbital in 2017, the most recent year reported, was limited to 15 municipalities with six (MI: 27.4%, IL: 21.3%, MO: 18.2%, Virgin Islands: 16.4%, PA: 7.7%, TX: 5.8%) accounting for the preponderance (96.9%).

## Discussion

This report identifies dynamic changes in individual opioid distribution rates in veterinary teaching institutions over the past 14 years. Opioids are a key component of anesthetic procedures and acute pain management, particularly for small animals (19). The central importance of opioids is complicated, however, by the unfortunate ongoing reality of the opioid epidemic including increasing number of overdoses and the diversion of prescription opioids for recreational use (1–3). A survey of 700 Veterinary Information Network (VIN) members found that almost all respondents report using buprenorphine in their practices (96.0%), most use hydromorphone (71.7%) and hydrocodone (61.4%), less than half used morphine (44.5%), one-quarter with fentanyl patches (27.3%), and only one out of nine used methadone (10.9%) (17). The present findings expand upon both the VIN study (17) and another ARCOS report that was limited to a single state (16). The temporal pattern for individual opioids is highly variable but there is evidence to suggest a decline in hydrocodone use (which preceded upscheduling from Schedule III to II), a recent decline in codeine, a decline in fentanyl use from 2013 to the present, and a gradual increase in methadone distribution. The decline in fentanyl since 2013 may be due in part to the implementation and modification of the FDA Risk Evaluation and Mitigation Strategy and the greater awareness of the risks of fentanyl-containing products. Unlike many opioids, the DEA production quota for methadone increased by 11.4% from 2010 until 2020 (10, 11). Overall, methadone was the predominant opioid in the US, accounting for twice the MME as the next most common agent, oxycodone (7). Among the subset of veterinarians who administered methadone, almost two-thirds (64.0%) report using it frequently (17).

The overall use of opioids by MME for veterinary purposes has been relatively stable from 2014 to 2019, with only a 5.7% reduction. This consistency is in contrast to the larger opioid pattern for human medical use in which there have been large overall declines (7) and also declines among specific agents like fentanyl (8). The DEA does impose production quotas and these have been limited for many opioids (11). Further study may consider continued monitoring of veterinary use of controlled substances as the DEA recently increased the production quotas by 15% for fentanyl, morphine, hydromorphone, and codeine (28).

A pronounced reduction in stimulants was noted. This was striking for amphetamine although the same pattern was observed for methylphenidate, lisdexamfetamine, and cocaine. Stimulants like amphetamine and methylphenidate are employed for hyperkinesis or Attention Deficit Hyperactivity Disorder in dogs (30). Methylphenidate may also be used for narcolepsy (31). Cocaine may have been replaced by other less restricted substances (e.g., phenylephrine) for the diagnosis of Horner's syndrome (32). Further research with other data sources would be necessary to characterize the indication(s) for which these other stimulants were used for, e.g., separation anxiety, obesity, narcolepsy, or anesthesia reversal, and their substitution with other agents. The decline in stimulants identified here is in marked contrast to the general pattern in human use with yearly elevations in lisdexamfetamine and amphetamine (33).

The amount of pentobarbital distributed in 2017 (43.9 kg) was over 20-fold higher than the total of all other controlled substances combined. Pentobarbital is a standard agent for euthanasia and may be an option for seizures that are refractory to other treatments (31). This increase in pentobarbital is of particular concern since veterinarians are more likely than the general population to die by suicide (34) with poisoning being the most common mechanism of death and pentobarbital the most frequent agent (6). This disturbing fact has prompted calls for improved pentobarbital storage practices (35).

Although a strength of this report is its national coverage using a database with uniform procedures, there are also some limitations. This investigation was limited to Schedule II and III substances. These categories include seven of the nine most used opioids (17) but not Schedule IV tramadol or butorphanol. Furthermore, this dataset is based on information submitted by manufacturers and distributors to the DEA and occasionally (e.g., pentobarbital circa 2010), this information should be interpreted cautiously. A non-negligible subset of the controlled substance distribution reported may be employed for research at veterinary institutions rather than direct animal care. Furthermore, the impact of veterinarians providing portable prescriptions upon request and those medications subsequently filled and distributed outside veterinary teaching institutions was not evaluated (i.e., brick and mortar as well as online pet pharmacies).

In conclusion, this report identifies an overall stability in opioid use among veterinary teaching institutions with decreases in some agents like hydrocodone, codeine, and more recently fentanyl as well as an increase in others (methadone). Also notable was the elimination of many controlled stimulants. Examination of individual states reveal a non-homogenous distribution pattern. Together, these findings note regionally restricted use of controlled substances by veterinary teaching institutions. Ongoing vigilance is warranted to encourage judicious use by the next generation of veterinarians.

## Data Availability Statement

The original data can be found at: https://www.deadiversion.usdoj.gov/arcos/retail_drug_summary/index.html. The datasets generated can be found in the Supplementary Material.

## Author Contributions

BP was responsible for study design, analysis, figure preparation, and writing an early draft. KM, LK, and PH contributed to data interpretation and revisions. All authors approved the final version.

## Conflict of Interest

BP is part of an osteoarthritis research team supported by Pfizer. The remaining authors declare that the research was conducted in the absence of any commercial or financial relationships that could be construed as a potential conflict of interest.

## Supplementary Material

Supplementary Figure 1 Controlled substance distribution by weight (grams) by year for 2001 to 2005 to US veterinary teaching institutions as reported to the Drug Enforcement Administration's Automation of Reports and Consolidated Ordering System. The peak year for distribution is shown in parentheses.
