# Supplementary figures and images for: Assessment of Controlled Substance Distribution to U.S. Veterinary Teaching Institutions From 2006 to 2019
Source: Front Vet Sci. 2020 Dec 18;7:615646. doi: 10.3389/fvets.2020.615646 (PMC7775551; doi:10.3389/fvets.2020.615646)

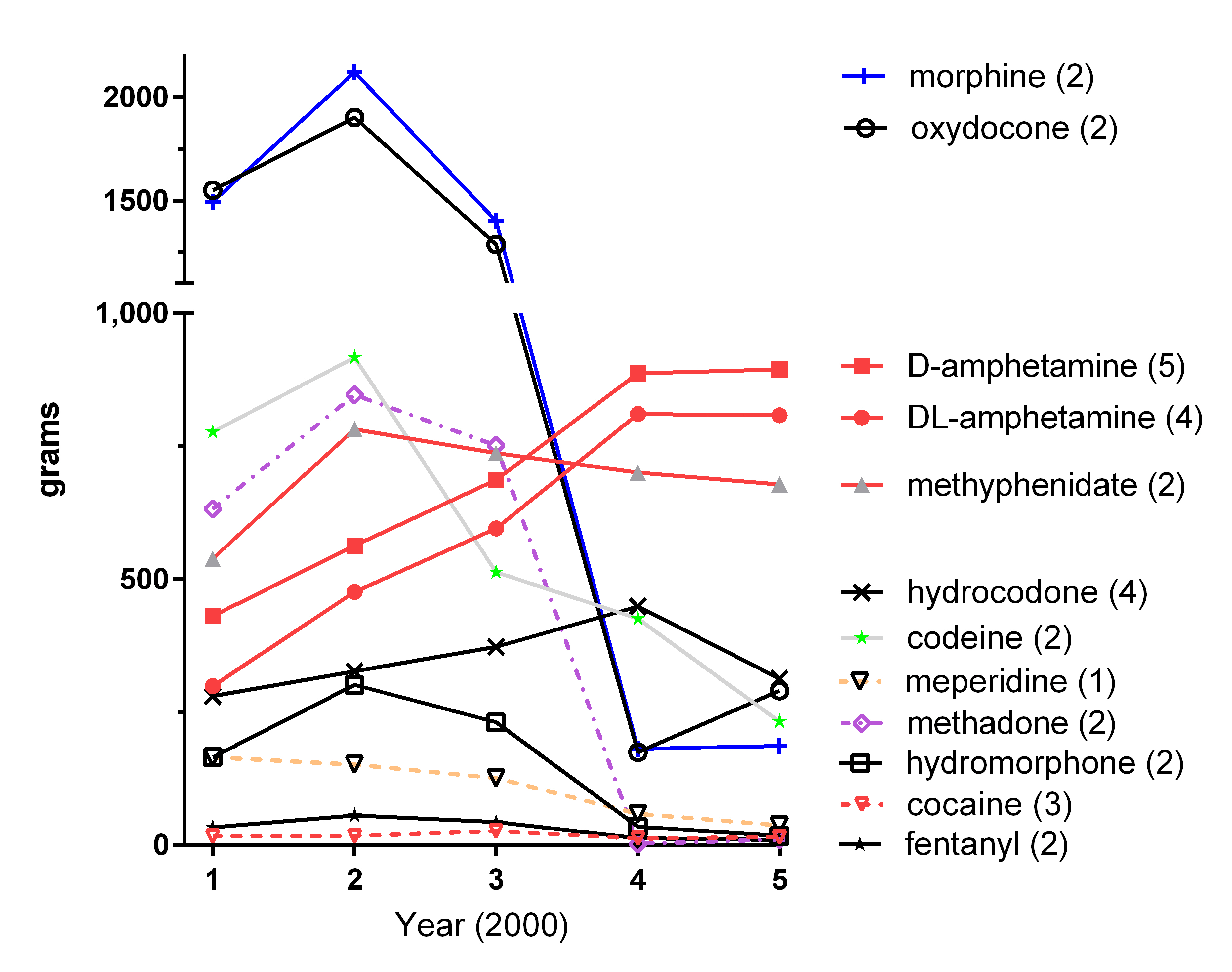

Supplement: Supplementary Figure 1 — Controlled substance distribution by weight (grams) by year for 2001 to 2005 to US veterinary teaching institutions as reported to the Drug Enforcement Administration's Automation of Reports and Consolidated Ordering System. The peak year for distribution is shown in parentheses. [file Image_1.JPEG]
